# Draft Genome Sequence of a Listeria monocytogenes Isolate of Core Genome Multilocus Sequence Typing Complex Type 2521 from Ready-to-Eat Meat Sausage Related to an Outbreak (Sigma1) in Germany

**DOI:** 10.1128/MRA.00267-20

**Published:** 2020-04-30

**Authors:** M. Adler, S. Lüth, S. Kleta, S. Al Dahouk

**Affiliations:** a German Federal Institute for Risk Assessment, Department of Biological Safety, Berlin, Germany; b Freie Universität Berlin, Institute of Biology, Berlin, Germany; c RWTH Aachen University Hospital, Department of Internal Medicine, Aachen, Germany; University of Maryland School of Medicine

## Abstract

We report here the draft genome sequence of a Listeria monocytogenes strain, core genome multilocus sequence typing (cgMLST) complex type 2521 (CT2521), isolated from ready-to-eat meat sausage related to a protracted and supraregional listeriosis outbreak (Sigma1) in Germany from 2014 to 2019.

## ANNOUNCEMENT

Listeria monocytogenes lives as a saprophyte in the soil but may enter the food chain through raw animal and vegetable products. In food production plants, the pathogen colonizes the food processing environment by establishing biofilms. Transmission of L. monocytogenes to humans occurs via consumption of contaminated food, especially prepackaged ready-to-eat products (1). Severe and fatal listeriosis cases appear mainly in vulnerable populations, such as pregnant women, newborns, and immunocompromised or older people (2).

Integrated molecular surveillance of clinical isolates defined a protracted and supraregional listeriosis outbreak cluster of core genome multilocus sequence typing (cgMLST) complex type 2521 (CT2521) (3) in Germany in 2014 to 2019. The outbreak, referred to as Sigma1, comprised 37 notified cases and peaked in 2018 (4). Follow-up controls at the implicated production facility yielded samples from various ready-to-eat meat products and food processing environments contaminated with L. monocytogenes cgMLST CT2521. CT2521 isolates belong to MLST clonal complex 8 and sequence type 8 (ST8), the most frequent ST among clinical isolates in Germany (5), and can be associated with bacteremia (6). Here, we provide the sequence of a representative food isolate (19-LI00774-0) of the outbreak cluster from ready-to-eat sausage.

The strain arrived at the National Reference Laboratory for Listeria monocytogenes as an agar swab of a food isolate and was cultivated on sheep blood agar overnight at 37°C with subsequent passage of a single colony onto fresh sheep blood agar. After overnight incubation at 37°C, bacterial cells were scraped off and lysed according to the Pulse Net standardized laboratory protocol for whole-genome sequencing of Gram-positive bacteria (https://www.cdc.gov/pulsenet/pdf/pnl32-miseq-nextera-xt.pdf). Genomic DNA was extracted with the QIAamp DNA minikit (Qiagen, Hilden, Germany) according to the manufacturer’s instructions. Sequencing libraries were constructed using the Nextera XT DNA sample preparation kit (Illumina, Inc., San Diego, CA, USA). Sequencing was performed in paired-end mode with 2 × 300-bp reads on the Illumina MiSeq sequencer using the MiSeq reagent v3 600-cycle kit (Illumina). A total of 1,474,622 reads were generated, and 1,431,826 trimmed reads were assembled using the Assembly-Based Quality Assessment for Microbial Isolate Sequencing (AQUAMIS; https://gitlab.com/bfr_bioinformatics/AQUAMIS) pipeline (trimming and read quality control, fastp version 0.19.5; classification, Kraken2 version 2.0.8_beta; assembly workflow, Shovill version 1.0.4; reference search, Mash version 2.1; assembly quality control, QUAST version 5.0.2).

Assembly yielded a total of 3,011,407 bp, resulting in 21 contigs, with a G+C content of 38.4%, an *N*_50_ value of 579,639 bp, and a coverage depth of 90×.

Ridom SeqSphere+ version 6.0.2 (Ridom GmbH, Münster, Germany) was used with default parameters to determine the cgMLST type. The assembled genome was submitted to GenBank and annotated with the Prokaryotic Genome Annotation Pipeline (PGAP; https://www.ncbi.nlm.nih.gov/genomes/static/Pipeline.html). Annotation returned 3,070 genes with 2,977 protein-coding sequences, 6 complete rRNAs, 7 incomplete rRNAs, 17 pseudogenes, 4 noncoding RNAs, and 59 tRNAs.

### Data availability.

This whole-genome shotgun project has been deposited at DDBJ/ENA/GenBank under the accession number JAANCL000000000. The corresponding raw reads are accessible under SRA number PRJNA607688.

## References

[1] Swaminathan B, Gerner-Smidt P 2007 The epidemiology of human listeriosis. Microbes Infect 9:1236–1243. doi:10.1016/j.micinf.2007.05.011. 17720602

[2] Schuchat A, Swaminathan B, Broome CV 1991 Epidemiology of human listeriosis. Clin Microbiol Rev 4:169–183. doi:10.1128/cmr.4.2.169. 1906370 PMC358189

[3] Ruppitsch W, Pietzka A, Prior K, Bletz S, Fernandez HL, Allerberger F, Harmsen D, Mellmann A 2015 Defining and evaluating a core genome multilocus sequence typing scheme for whole-genome sequence-based typing of *Listeria monocytogenes*. J Clin Microbiol 53:2869–2876. doi:10.1128/JCM.01193-15. 26135865 PMC4540939

[4] Wilking H, Lachmann R, Holzer A, Boone I, Halbedel S, Flieger A, Stark K 2019 Listeriose-Ausbruch mit *Listeria monocytogenes* sequenz-cluster-typ 2521 (Sigma1) in Deutschland. Epidemiologisches Bull 41:431–432. doi:10.25646/6308.2.

[5] Halbedel S, Prager R, Fuchs S, Trost E, Werner G, Flieger A 2018 Whole-genome sequencing of recent *Listeria monocytogenes* isolates from Germany reveals population structure and disease clusters. J Clin Microbiol 56:e00119-18. doi:10.1128/JCM.00119-18. 29643197 PMC5971532

[6] Maury MM, Tsai YH, Charlier C, Touchon M, Chenal-Francisque V, Leclercq A, Criscuolo A, Gaultier C, Roussel S, Brisabois A, Disson O, Rocha EPC, Brisse S, Lecuit M 2016 Uncovering Listeria monocytogenes hypervirulence by harnessing its biodiversity. Nat Genet 48:308–313. doi:10.1038/ng.3501. 26829754 PMC4768348

